# The variation in the self-perceived quality of life and health care amongst smokers, passive smokers, ex-smokers and non-smokers in Canada

**DOI:** 10.1186/1753-6561-9-S7-A18

**Published:** 2015-10-27

**Authors:** Samy Beshay, Hany Beshay

**Affiliations:** 1 Royal College of Surgeons In Ireland, Dublin, Ireland; 2 Terry Fox Medical Centre, Mississauga, Ontario, Canada

## Background

In 2012 nearly 20% of Canadians aged 12 and above had stated they smoked tobacco frequently, costing the health care system over $4.4 billion in health related illnesses. The aim of this study was to assess degrees of tobacco inhalation of smokers, non-smokers, ex-smokers, passive smokers and current smokers and their perceived quality of life and health.

## Methods

The survey was conducted in the waiting room of two medical walk-in-clinics. The questionnaire comprised of four main aspects including age of the patient, identify themselves as a frequent smoker, a non-smoker (passive) who is regularly exposed to smoke, a past (ex-) smoker and a non-smoker who is not regularly exposed to tobacco smoke. Valid consent was obtained from the patients and patients under the age of 18 were not included in the study.

## Results

A total of 387 patients completed the survey including 198 non-smokers, 83 passive smokers, 51 ex-smokers and 55 current smokers. The oldest group was the ex-smokers of a mean age of 52.6 years and the youngest was the smokers at 36.6 years (p < 0.001). In between were the passive smokers at 43.6 years and non-smokers at 48.2 years (p= 0.002).

## Conclusion

This research found that current smokers have a persistently lower self-reported quality of life and health care as compared with the other groups. It is also evident that patients who quit smoking do not suffer a loss in quality of life nor health compared to non-smokers. In addition, this research indicates that smoking not only impacts a patient's health, but their overall QoL as well.

